# Public perception and correlates of Mpox vaccine acceptability in northern Nigeria: a mixed methods study

**DOI:** 10.11604/pamj.supp.2025.50.1.44316

**Published:** 2025-03-03

**Authors:** Zubairu Iliyasu, Hadiza Musa Abdullahi, Amina Abdullahi Umar, Tahir Dahiru, Fatimah Ismail Tsiga-Ahmed, Taiwo Gboluwaga Amole, Aminatu Ayaba Kwaku, Abubakar Mohammed Jibo, Humayra Aisha Bashir, Hamisu Muhammed Salihu, Muktar Hassan Aliyu

**Affiliations:** 1 Division of Epidemiology and Biostatistics, Department of Community Medicine, Bayero University Kano, Kano, Nigeria,; 2 University of Oxford, Oxford, United Kingdom,; 3 Kano Independent Research Centre Trust, Kano, Nigeria,; 4 Vanderbilt Institute for Global Health, Vanderbilt University Medical Center, Tennessee, United States of America

**Keywords:** Mpox, vaccine acceptance, predictors, hesitancy, Nigeria

## Abstract

**Introduction:**

little has been documented about public perceptions and acceptance of the mpox vaccine in endemic countries, such as Nigeria. We assessed public awareness, risk perception, and correlates of vaccine acceptability in urban Kano, Nigeria.

**Methods:**

employing a mixed methods design, structured questionnaires were administered to 415 adults in metropolitan Kano. The data were analyzed using binary logistic regression and the framework approach.

**Results:**

nearly all respondents (99.0%, n=411) have heard about mpox, but only one-half (49.9%, n=207) of the respondents were willing to take the mpox vaccine. Female respondents were 33% less likely to accept mpox vaccination (adjusted odds ratio, aOR=0.67, 95% confidence interval CI: 0.42-0.92). Respondents who perceived mpox as a severe disease (aOR = 1.37, 95% CI, 1.12-2.20), those who self-assessed as high risk (aOR=2.10, 95% CI, 1.10-7.46) or who perceived vaccines as protective (aOR=8.99, 95% CI,1.79-45.30) were more likely to accept the vaccine. There were three-fold increased odds of accepting the mpox vaccine among participants who received vaccinations during adulthood (aOR=3.58, 95% CI, 2.20-5.82), and those who were vaccinated against COVID-19 (aOR=2.86, 95% CI, 1.45-5.64). Reasons for vaccine hesitancy include vaccine safety concerns, low-risk perception, mistrust of authorities and pharmaceutical companies, the newness of the vaccine, hurried introduction, fear of side effects, and perceived misplaced government priorities.

**Conclusion:**

Mpox awareness was near-universal in our study population, but mpox vaccine acceptance was sub-optimal. mpox vaccine acceptance was influenced by sociodemographic (gender, ethnicity, religion), vulnerability (perceived risk, perceived effectiveness of vaccines), and health behavior-related (COVID-19 vaccination status) factors. Risk communication, community engagement, and socio-behavioral interventions could help build public confidence and boost vaccine acceptance in similar settings.

## Introduction

Mpox is a zoonotic disease intricately linked to smallpox. The disease was recently renamed mpox, and it is caused by a double-stranded DNA virus belonging to the Orthopoxvirus of the Poxviridae family [1]. The mpox virus was first detected in imported monkeys in Denmark in 1958 [2] and subsequently in a 9-year-old boy in the Democratic Republic of Congo (DRC) in 1970 [3]. Interaction with infected wildlife, peri-domestic rodents and squirrels, and bushmeat trade are primary sources of human infection [4]. Human-to-human transmission follows contact with patients, body fluids, respiratory droplets, sexual contact, and vertical transmission. The clinical features of mpox are similar but milder compared to smallpox. After a 5 to 21-day incubation period, infected persons typically develop fever, generalized headache, enlarged cervical and inguinal lymph nodes, fatigue, back pain, myalgia, and itchy painful centrifugal rashes [5,6]. During the current global outbreak, however, novel features have been reported, including genital rash, severe proctitis, urethritis, and urinary retention in men who have sex with men [7]. Most cases are self-limiting, but about 40% of affected people develop complications including rectal pain, odynophagia, penile oedema, skin and anorectal abscesses, septicemia, encephalitis, myocarditis, bronchopneumonia, corneal ulcers, and blindness [8]. Abortion and stillbirth have also been reported among pregnant women [9]. The case fatality rate ranges from 7% to 10% [8].

Mpox is endemic in Central and West Africa, including Nigeria since the late 1970s. Before the recent global outbreak, from 2018 to 2021, six African countries reported human cases of mpox with more than 18,000 suspected cases in the Democratic Republic of Congo alone [10]. Sporadic cases were also reported in the Western hemisphere in 2003, acquired from imported small mammals co-housed with prairie dogs. Barely recovering from an unprecedented COVID-19 pandemic, cases of mpox spread outside the endemic regions, raising concerns about another disruptive pandemic. By the 30th of January 2023, over 85,000 confirmed cases and 89 deaths were reported to the World Health Organization from 110 countries [11]. While most cases in the recent outbreak in non-endemic countries affected MSM, cases were also reported among children, young adults, the elderly, and women [12]. Two of the cases in the UK had a recent travel history to Nigeria while one was a health worker who managed one of the cases [13]. However, most cases had no links with endemic countries. To ensure coordinated global solidarity, the WHO declared the outbreak a public health emergency of international concern on the 23rd of July 2022 [11]. The US followed suit by declaring mpox a public health emergency on the 4th of August 2022.

Nigeria is one of the mpox endemic West African countries [14]. Historically, the largest outbreak in Nigeria occurred in 2017, with 197 suspected and 68 confirmed cases, and between September 2017 and June 2022, a total of 622 suspected cases of mpox were reported from 33 states, including Kano, the most populous state in Nigeria [15]. This study aims to assess awareness, sources of information, risk perception, and correlates of the mpox vaccine’s among adults in metropolitan Kano.

## Methods

**Study area and population:** the study was conducted in two metropolitan local government areas (LGAs), Nasarawa and Tarauni in Kano, in northern Nigeria. The projected populations of Tarauni and Nasarawa LGAs were 971,043 and 360,261, respectively [16]. The main occupations of inhabitants are trading, entrepreneurship, civil service, and farming. The study population consisted of adult men and women (≥18 years) resident in Kano for at least 6 months. Visitors, persons who withheld consent, and those with cognitive impairment were excluded.

**Study design and sampling:** a mixed methods sequential, explanatory design was used with a pragmatic paradigm. The sample size was estimated using Fisher´s formula [17], mpox acceptance from a previous study (50%) [18], 95% confidence level, and 5% error margin. The calculated minimum sample size (385) was increased by 10% to address non-response and rounded up to 450.

A multistage sampling method was used. In the first stage, half of the ten wards in each of the two LGAs were sampled using a simple ballot. In the second stage, one settlement was selected from each sampled ward using the same method. Numbers were then allotted to the selected settlements proportionate to their populations. After household enumeration and sampling interval determination, systematic sampling was used to select respondents in each settlement. The first household was selected by simple ballot between households whose serial number was 1 and the one whose serial number corresponded with the settlement´s sampling interval. Subsequent households were identified by adding the sampling interval to the previously studied household´s serial number. Finally, within each sampled household, eligible adults consented after a detailed explanation of the study objectives. When more than one eligible respondent was found in a household, only one was selected by simple ballot and interviewed.

**Measures and data collection:** the survey questionnaire was adapted from previous studies [19,20]. The first section documented sociodemographic characteristics, including age, sex, marital status, ethnicity, education, religion, occupation, parity, history of a chronic medical disorder, and self-assessed health status. The second section inquired about awareness of mpox, sources of information, messages received, perceived causes, sources of infection, species affected, transmission, and symptoms. The third section determined self-perceived mpox risk using the question “How would you assess your chance of getting mpox? The responses were (‘high’ or ‘low’), whether or not the respondent was worried about getting mpox, perceived severity, and whether the respondent knew someone who had mpox. The fourth section determined mpox vaccine acceptability by asking, ‘If the mpox vaccine is made available, will you take it? Response options include ‘I will take it’, ‘I am not sure’ and ‘I will not take it’.

To determine the psychometric properties (re-validation, reliability, and consistency) of the adapted questionnaire, a pretest was carried out on a 10% sample of the questionnaires in another community (Gwale LGA, Kano, Nigeria). All scales were reliable and sections were consistent with Cronbach’s alpha of at least 0.80. To elaborate on the survey responses, twenty survey respondents were purposively selected and interviewed in-depth. These semi-structured interviews explored motivations for vaccine acceptance and reasons for hesitancy or ambivalence. There were probes for nuanced descriptions. Sample stratification was based on profession and gender.

The study protocol was reviewed and approved by the Kano State Ministry of Health research ethics committee. Using the local Hausa language, trained research assistants informed eligible persons in sampled households about the study objectives, eligibility criteria, sampling process, and procedure. Participants were also informed that involvement was voluntary and that withholding consent had no consequences. Literate persons provided signed informed consent, while their non-literate counterparts thumb-printed the consent form before the interviews. Interviews were conducted face-to-face, in the respondents´ homes, away from other family members. Completed questionnaires were checked in the field by the supervisors and independently entered into a password-protected database by data clerks at Aminu Kano Teaching Hospital. Research staff received training on establishing rapport, obtaining informed consent, protecting human research participants, and conducting interviews.

**Statistical analysis:** data were analyzed using SPSS Version 27 (IBM Corp., Armonk, NY). Numeric variables were summarized using means, standard deviation, or median and range. Categorical variables were presented as frequencies and percentages. Pearson´s Chi-square or Fisher´s exact test was used to test the association between sociodemographic (respondent´s sex, ethnic group, religion, number of children, occupation, perceived severity of mpox, mpox risk perception, vaccines protect against diseases, have received a vaccine in adulthood, vaccinated against COVID-19) and the primary outcome, (willingness to be vaccinated against mpox) [21]. Type I error was fixed at 5% for all tests. Binary logistic regression model was used to determine the independent predictors of willingness to be vaccinated against mpox. A stepwise logistic regression model included independent variables with p<0.10 at the bivariate level [22]. Adjusted odds ratios (aORs) and their 95% confidence intervals (CIs) were used to measure the strength and direction of the effect of the independent variables on the outcome. Hosmer-Lemeshow statistic and Omnibus tests were used to determine model fitness, with a Hosmer-Lemeshow chi-square yielding a p-value of >0.05 considered a good fit [23].

**Qualitative data analysis:** qualitative interviews were recorded and transcribed verbatim. Thematic analysis was performed based on the ‘Framework Approach’ [24], and included familiarization through repeated reading, coding, theme generation, applying the codes to the transcripts, matrix formation, and interpretation. Findings from the two components of the mixed-methods study were integrated [25].

## Results

Of the 450 people invited, (92.2%, n=415) completed the interviews. Most respondents were female (62.2%, n=258), of Hausa-Fulani ethnicity (91.6%, n=380) and Muslim (96.6%, n=401). Respondents´ mean age (± standard deviation (SD) was 31.9 ± 10.78 years, and the majority (88.7%, n=368) had at least secondary education. Over one-tenth (13.5%, n=56) had a chronic medical illness and one-fifth (21.0%, n=87) were vaccinated against COVID-19 (Table 1). The majority of respondents (94.9%, n=394) considered vaccines to be protective, and (43.9%, n=182) had received a vaccine as adults. Further, about half (51.6%, n=214) of the respondents had fully immunized or up-to-date children.

**Table 1:** sociodemographic characteristics of adult respondents, Kano, Nigeria, 2022

Characteristics	Frequency; N=415
**Sex**	**No. (%)**
Male	157 (37.8)
Female	258 (62.2)
**Age group**	
<30	206 (49.6)
30-39	123 (29.6)
40-49	48 (11.6)
≥50	38 (9.2)
**Ethnicity**	
Hausa/Fulani	380 (91.6)
Others*	35 (8.4)
**Religion**	
Islam	401 (96.6)
Christianity	14 (3.4)
**Marital status**	
Married	305 (73.5)
Single/divorced/widowed	110 (26.5)
**Number of children**	
0	140 (34.0)
1	33 (8.0)
2-4	142 (34.5)
≥5	97 (23.5)
**Youngest child’s immunization status**	
Fully immunized/up-to-date	214 (51.6)
Partially immunized/not immunized	47 (11.3)
Not applicable	154 (37.1)
**Education**	
Non-Formal	30 (7.2)
Primary	17 (4.1)
Secondary	178 (42.9)
Post-secondary	190 (45.8)
**Occupation**	
Unemployed/student	105 (25.3)
Trading/farming	130 (31.3)
Civil service	100 (24.1)
Business	74 (17.8)
Others	6 (1.5)
**Monthly income (Naira)**	
<30,000	254 (61.2)
30,000-99,000	143 (34.5)
≥100,000	18 (4.3)
**History of chronic medical disorder**	
Yes	56 (13.5)
No	359 (86.5)

Others*=Yoruba, Igbo, Nupe, Igala, Sayawa, Egbira, Kanuri; Others**= Driver, Seamstress, Restauranteur, Hair Saloon

**Awareness of mpox, risk perceptions and sources of information:** nearly all respondents (99.0%, n=411) have heard about mpox, mainly on radio (89.6%), social media (40.2%), television (18.3%), and from healthcare workers (14.5%). Messages received include sources of infection (45.5%), mode of spread (47.2%), symptoms (53.3%), and prevention (29.9%). The majority of respondents indicated that mpox was caused by a virus (60.7%, n=252), others mentioned bacteria (12.8%, n=53), and witchcraft/evil spirits (0.96%, n=4). Most respondents (81.0%, n=336) indicated that mpox affects humans, with (15.2%, n=63) mentioning monkeys. Transmission methods mentioned include consumption of monkey/bush meat (48.2%), contact with infected animals (24.1%), and patients (17.6%). A few respondents (1.4%) considered the possibility of sexual transmission. Over one half of the respondents (52.8%) felt mpox is a serious disease, 45.1% were worried, but only 3.6% self-assessed a high risk (Table 2).

**Table 2:** monkeypox awareness, risk perception and vaccine acceptability, Kano, Nigeria (N=415)

	No. (%)
**Awareness of causes, transmission, and symptoms**	
Have heard of monkeypox	411 (99.0)
**Source(s) of information**	
Radio	371 (89.6)
Social media	167 (40.2)
Television	76 (18.3)
Health care worker	60 (14.5)
Family members/friends	44 (10.6)
Newspaper	29 (7.0)
Others*	18 (4.3)
**Perceived causes of monkeypox**	
Stated that a virus causes monkeypox	252 (60.7)
Indicated bacteria as a cause of monkeypox	53 (12.8)
Witchcraft/sorcery/evil spirit	4 (1.0)
Did not know the cause of monkeypox	106 (25.5)
**Monkeypox affects the following**	
Humans	336 (81.0)
Monkeys	63 (15.2)
All animals	9 (2.1)
Don’t know	7 (1.7)
**Main methods of transmission**	
Eating monkey meat/bush meat	200 (48.2)
Touching infected animals	100 (24.1)
Contact with body fluid from an infected person	73 (17.6)
Eating improperly cooked meat	15 (3.7)
Sexual contact	6 (1.4)
Contact with infected surfaces	4 (0.96)
Don’t know	17 (4.1)
**Symptoms mentioned (multiple responses)**	
Fever	405 (97.6)
Rash	389 (93.7)
Body pains	376 (90.6)
Swollen lymph nodes	247 (59.5)
Genital rash	183 (44.1)
Others**	2 (0.48)
Knows someone who had monkeypox	45 (10.9)
**Monkeypox risk perception**	
Considers monkeypox a serious disease	219 (52.8)
Worried about contracting monkeypox	187 (45.1)
Feels his/her chance of getting monkeypox is high	15 (3.6)
Believes vaccines protect against diseases	394 (94.9)
Has received a vaccine during adulthood	182 (43.9)
Has received recommended doses of the COVID-19 vaccine	87 (21.0)
**Willingness to be vaccinated**	
Willing to receive the monkeypox vaccine	207 (49.9)
Unwilling to take the monkeypox vaccine	52 (12.5)
Not sure/undecided	156 (37.6)
**Reasons for unwillingness/indecision+ (n=208)**	
I am concerned about vaccine safety	116 (55.8)
I don’t think I can get monkeypox	41 (19.7)
I don’t trust the authorities/pharmaceutical companies	33 (15.9)
Monkeypox is not serious enough to require vaccination	28 (13.5)
Pregnancy/breastfeeding	20 (9.6)

Others*= worship center, market, abbatoir; Others**=redness of the eyes, sore throat; + Multiple responses

**Willingness to accept mpox vaccination and reasons for hesitancy:** half of the respondents (49.9%, n=207) were willing to take the mpox vaccine, about one-third (37.6%, n=156) were undecided and the rest (12.5%, n=52) were unwilling. Of those that were unwilling or undecided (n=208), over one-half (55.8%, n=116) were concerned about vaccine safety, (19.7%, n=41) self-assessed a low risk, (15.9%, n=33) had little trust in the authorities and pharmaceutical companies, and (13.5%, n=28) did not consider mpox a threat (Table 2).

**Predictors of mpox vaccine acceptability:** bivariate analysis showed an association between mpox vaccine acceptance and gender, ethnicity, religion, and occupation. In addition, vaccine acceptance was associated with perceived severity, risk perception, belief in the effectiveness of vaccines, vaccination during adulthood, and COVID-19 vaccination (p<0.05). At the multivariate level, the same factors independently predicted mpox vaccine acceptance, except occupation. Specifically, female respondents were 33% less likely to accept mpox vaccination (adjusted odds ratio, aOR = 0.67, 95% confidence interval CI: 0.42-0.92). In contrast, non-Hausa/Fulani respondents had 56% (aOR = 1.56, 95% CI, 1.10-3.85) increased likelihood of accepting the mpox vaccine. Likewise, non-Muslim respondents had almost six-fold increased odds of vaccine acceptance (aOR = 5.70, 95% CI, 1.14-37.79). In addition, respondents who perceived mpox as a severe disease (aOR = 1.37, 95% CI, 1.12-2.20) had 37% increased likelihood of vaccine acceptance, while a high self-assessed risk (aOR = 2.10, 95% CI, 1.10-7.46) doubled the odds of vaccine acceptance. Further, respondents who perceived vaccines as protective (aOR = 8.99, 95% CI, 1.79-45.30) and received vaccination during adulthood (aOR = 3.58, 95% CI, 2.20-5.82) had nine- and three-fold increased odds of accepting mpox vaccine. Finally, respondents vaccinated against COVID-19 (aOR = 2.86, 95% CI, 1.45-5.64) had nearly three-fold increased probability of accepting mpox vaccine (Table 3).

**Table 3:** logistic regression model for predictors of monkeypox vaccine acceptability among adults, Kano, Nigeria (N=415)

Characteristics	N	Willing to accept monkeypox vaccination; No. (%)	P-value	Crude OR (95% CI)	Adjusted OR (95% CI)	P-value
**Sex**			0.03*			
Male	157	89 (56.7)		Referent	Referent	
Female	258	118 (45.7)		0.64 (0.43-0.96)	0.67 (0.42-0.92)	0.04*
**Age group**			0.48			
<30	206	97 (47.1)		--	--	
30-39	123	61 (49.6)		--	--	
40-49	48	28 (58.3)		--	--	
≥50	38	21 (55.3)		--	--	
**Ethnicity**			0.021*			
Hausa/Fulani	380	183 (48.2)		Referent	Referent	
a) Others^a^	35	24 (68.6)		2.35 (1.12-4.93)	1.56 (1.10-3.85)	0.024*
**Religion**			0.006*			
Islam	401	195 (48.6)		Referent	Referent	
Christianity	14	12 (85.7)		6.34 (1.40-28.68)	5.70 (1.14-37.79)	0.016*
**Marital status**			0.25			
Married	305	60 (54.6)		--	--	
Single/widowed/divorced	110	147 (48.2)		--	--	
**Number of children**			0.059			
0	143	74 (51.8)		Referent	Referent	
1	33	9 (27.3)		0.35 (0.15-0.80)	0.51 (0.20-1.31)	0.58
2-4	142	75 (52.8)		1.04 (0.66-1.66)	1.17 (0.62-2.19)	0.09
≥5	97	49 (50.5)		0.95 (0.57-1.59)	1.24 (0.62-2.48)	0.91
**Youngest child’s immunization status**			0.57			
Fully immunized/up to date	214	102 (47.7)		--	--	
Partially immunized/not immunized	47	23 (48.9)		--	--	
Not applicable	154	82 (53.3)		--	--	
**Education**			0.61			
No Formal	30	12 (40.0)		--	--	
Primary	17	10 (58.8)		--	--	
Secondary	178	88 (49.4)		--	--	
Post-secondary	190	97 (51.1)		--	--	
**Occupation**			0.015*			
Unemployed/student	105	52 (49.5)		Referent	Referent	
Trading/farming	130	57 (43.9)		0.80 (0.48-1.33)	0.81 (0.40-1.61)	0.68
Civil service	100	62 (62.0)		1.66 (0.95-2.90)	1.18 (0.57-2.44)	0.54
Business	74	31 (41.9)		0.73 (0.40-1.34)	0.50 (0.22-1.14)	0.79
b) Others^b^	6	5 (83.3)		5.09 (0.58-45.12)	1.57 (0.15-15.93)	0.31
**Monthly income (Naira)**			0.55			
<30,000	254	123 (48.4)		--	--	
30,000-99,000	143	73 (51.1)		--	--	
≥100,000	18	11 (61.1)		--	--	
**History of chronic medical disorder**			0.76			
Yes	56	29 (51.8)		--	--	
No	359	178 (49.6)		--	--	
**Perceived severity of monkeypox**			0.021*			
Low	196	86 (43.9)		Referent	Referent	
High	219	121 (55.3)		1.58 (1.07-2.33)	1.37 (1.12-2.20)	0.041*
**Monkeypox risk perception**			0.02*			
Low	395	192 (48.6)		Referent	Referent	
High	20	15 (75.0)		3.17 (1.13-8.90)	2.10 (1.10-7.46)	0.037*
**Vaccines protect against diseases**			<0.001*			
Yes	394	205 (52.0)		10.30 (2.37-44.83)	8.99 (1.79-45.30)	0.006*
No/Not sure	21	2 (9.5)		Referent	Referent	
**Have received a vaccine in adulthood**			<0.001*			
Yes	182	131 (72.0)		5.31 (3.47-8.11)	3.58 (2.20-5.82)	<0.001*
No	233	76 (32.6)		Referent	Referent	
**Vaccinated against COVID-19**			<0.001*			
Yes	87	70 (80.5)		5.74 (3.23-10.19)	2.86 (1.45-5.64)	0.003*
No	328	137 (41.8)		Referent	Referent	

a) Others=Yoruba, Igbo, Nupe, Igala, Sayawa, Egbira, Kanuri; b) Others = Driver, Seamstress, Restauranteur, Hair Salon; *Significant at p<0.05; OR: Odds Ratio, CI: confidence interval; Hosmer-Lemeshow Chi-square=8.65, p=0.37; the logistic model includes the following variables: sex, ethnic group, religion, number of children, occupation, perceived severity of monkeypox, monkeypox risk perception, vaccines protect against diseases, have received a vaccine in adulthood, vaccinated against COVID-19

**Qualitative findings:** in-depth interview themes included perceptions about mpox disease, transmission, the possibility of sexual transmission, high-risk groups, perceived severity, risk perception, precautions, willingness to take the vaccine, and reasons for vaccine hesitancy.

**Perceptions of mpox:** participants considered mpox a simulation of smallpox, and unlike smallpox, mpox heals without scars. They also indicated that mpox is a viral disease affecting mostly children, the elderly, those living in unhygienic environments, and those in occupations involving handling animals and bush meat. Others stated that mpox is acquired from eating leftover fruits and drinking water from monkey-frequented ponds.

“I think mpox is an imitation of smallpox (Hausa: Agana) and heals without scars. In contrast, smallpox leaves permanent scars on the face (Hausa: zanzana), making it easy to distinguish between the victims of the two diseases after recovery. Earlier we thought these diseases were caused by evil spirits, but the message we hear these days is that they are caused by germs,” man, 51-year-old.

“Mpox (Hausa: Farankaman birai) is caused by a virus that affects children, older people, and those engaged in occupations that involve close contact with animals (such as hunters, butchers and herdsmen). It is acquired through close contact with monkeys, eating or dealing in bush meat, and living in crowded unhygienic environments, particularly in rural areas,” woman, 43-year-old.

**Transmission of mpox:** some participants indicated that mpox is transmitted through close contact, body fluids, and sharing beddings and personal effects with patients, and through the respiratory route. Others mentioned eating leftover food and contaminated water while others entertained the possibility of a supernatural source.

“Mpox is transmitted by monkeys to humans when they (humans) drink water from ponds frequented by monkeys or when they eat fruits and other leftover food eaten by monkeys. Sharing beddings and clothes with a patient afflicted with mpox, and sharing bathing sponges and soap can transmit the infection. It can also be gotten from breathing close to those affected and living in crowded rooms,” man, 30-year-old.

“First and foremost, all illnesses including mpox are from God. In addition, when you eat fruits or food half eaten by monkeys, or you drink from the same ponds where sick monkeys drank, you can get mpox. It is also caused by living in an untidy environment, eating bush meat, and partaking in bush meat from sick animals, including chicken,” woman, 26-year-old.

**Sexual transmission:** though participants indicated that mpox was not one of the recognized sexually transmitted infections in the community, participants were of the view that sexual transmission could occur due to intimate body contact during such encounters.

“Yes, people are at risk of getting infected with mpox sexually. For instance, because of financial difficulties and poverty, women involved in the commercial sex business come into intimate contact with many people. If any of these clients have mpox, she will contract it and spread it to her new customers,” man, 30-year-old.

“I cannot say if mpox can be transmitted through sex. It is not among the diseases we know that are sexually transmitted. The indisputable ones are gonorrhea, syphilis, and HIV. But I can imagine, since sexual contact involves intimacy, it is possible. Though I have not heard of any such cases in this community,” woman, 26-year-old.

**Perceived severity:** participants consider mpox a serious disease and that it could be severe. Though compared to smallpox, mpox was seen as milder. Nonetheless, participants advised that measures should be promptly taken to control the disease.

“Mpox is a serious health problem. It is contagious and can make people very sick, but it is not as deadly as smallpox. Health authorities should not handle it with levity as they initially did with COVID-19. It should be taken seriously before it gets out of hand,” woman, 35-year-old.

“Mpox can be severe and easily spreads like other infectious diseases. It is a serious health problem, like COVID-19, spreads easily and is a major concern in this community because people travel from other parts of this country for business,” man, 45-year-old.

**Perceived high-risk groups:** participants identified hunters, cattle herders, fishermen, animal handlers, and health workers as the most at-risk groups. Regarding age groups affected, children, and the elderly were considered more vulnerable because of immaturity and immunosenescence, respectively.

“People at high risk of mpox include hunters, cattle herders, and fishermen. People who come in contact with animals such as veterinarians, butchers, zoo keepers, and farmers. Also, healthcare workers are at risk of getting infected by their patients,” woman, 28-year-old.

“Children, young people, HIV and diabetic patients, and the elderly are at high risk of contracting mpox. The elderly, because of their frailty, diminishing immunity, interaction with different kinds of people, and healthcare workers. Also, villagers who eat leftover mangoes eaten by monkeys without washing them can get infected,” woman, 38-year-old.

**Risk perception:** some participants justified their low-risk perception since they are not hunters, butchers, or bushmeat traders. In contrast, those engaged in these trades self-assessed their risk of acquiring mpox as high.

“No, I am not at risk of getting infected with mpox, because I don´t have any relationship with animal hunters nor do I eat or engage in the bush meat trade,” man, 42-year-old.

“Yes, of course, I am at risk of mpox because I interact with so many people, and being a butcher, my job entails handling animals, slaughtering them, and selling bush meat. Who knows, some of the animals could be infected and transmit the disease to me. God help us,” man, 39-year-old.

**Encounters with affected persons:** a participant narrated his encounter with a suspected case of mpox during a business trip to the central parts of Nigeria. Another participant recounted his experience with a suspected case two years earlier.

“I encountered a likely case of mpox in Ude village in Benue State during a business trip. In that community, roasted bush meat is a popular delicacy usually accompanied by a local alcoholic brew. In the market, I came across a person who had rashes all over his body and people avoided him. Some practically ran away from him and I also joined them for my safety. When I enquired about the man´s disease, they told me, it was mpox and that the man was a hunter who also engages in the preparation and sale of monkey meat,” man, 42-year-old.

“Yes, I came across a person infected with mpox in our community, about two years ago. He had rashes all over his body. He is now completely healed. The rashes disappeared without scars unlike those of smallpox patients years ago,” man, 31-year-old.

**Precautions against mpox:** participants indicated that they avoided persons with suspected lesions and those whose occupation involves handling animals. They also avoided bush meat and used face masks in crowded places.

“One of the precautionary measures I take is not to come in close contact with a person with skin eruptions, and people whose jobs entail hunting, handling animals, including monkeys, and I do not go to places where bush meat is sold. I always wear a face mask in public places,” man, 42-year-old.

“To avoid mpox, I distance myself from persons suspected to have mpox. Secondly, I used to maintain a clean environment, wash my hands regularly, use a facemask, avoid sickly pets and animals, eat a well-balanced diet, and drink clean water,” man, 39-year-old.

**Willingness to take mpox vaccine:** some participants were willing to take the vaccine unperturbed by rumors regarding the safety or side effects. They would also encourage their family to take the vaccine as they did during the COVID-19 pandemic.

“Yes, I am willing, like I told you, if the vaccine is available now, I would be the first person to take it to protect myself and my family from getting mpox. When the COVID-19 vaccine came, I and my family were among the first to take the vaccine regardless of the baseless rumors and we have been vindicated,” man, 39-year-old.

“I am ready and willing to be vaccinated against mpox disease, with all my family members. I know it will prevent us from getting infected with mpox. We are not disturbed by the stories and rumors being peddled about vaccines. We have confidence in the health workers and they mean well,” man, 31-year-old.

### Reasons for vaccine hesitancy

Some participants were unwilling to take the mpox vaccine for a variety of reasons. These include the newness of the vaccine, inadequate information safety, hesitancy among some health workers, and the hurried introduction of new vaccines compared to the older well-tested ones. Others were afraid of side effects and related it to the experience with COVID-19 vaccines and rumors of deaths on social media.

“You know this mpox vaccine you are talking about is new to us. We are not well-informed about it. Though many of us trust healthcare workers, even the HCWs appear not to be convinced as some of them even reject polio vaccines. they are unable to give us sufficient information about the safety of the vaccine. So, this is the problem. We do not want this hurried production of new vaccines. We trust older vaccines like the smallpox vaccine, measles, and BCG which have been in use for a long time,” man, 39-year-old.

“We are afraid of the side effects of the vaccine. For instance, when some people took the COVID-19 vaccine, they had a fever and couldn´t use their hands for some days. There are also reports of deaths on social media. Though we believe that healthcare workers will not deliberately harm us. However, we are scared of the unknown effects of the new vaccines, which the HCWs also do not seem to know. So, we want them (HCWs) to take the vaccine first. If nothing happens to them, then we will take it,” man, 45-year-old.

Other participants were unwilling to take the mpox vaccine (which they perceive authorities are interested in) in protest against their poor economic situation, hunger, poverty, and perceived neglect of the real problems bedeviling their society in preference for some foreign agenda. They also indicated that mpox cases are few and therefore not a priority.

“I am not ready to take any mpox vaccine because we have no food and no medicines for real diseases like malaria, cough, and other illnesses. Instead of solving these problems, the health authorities and government focus on an imaginary disease (mpox) that most of the members of the community have not seen or even heard about,” woman, 42-year-old.

“I am not interested in taking the mpox vaccine because it is all global politics. If not, why not provide free food, and antimalarials, our genuine needs, instead, they are spending our money on a vaccine for a disease that many people are not even aware of. It is not our problem, for now, let´s concentrate on our immediate challenges,” man, 47-year-old.

Some participants view the emphasis on vaccines for diseases such as mpox as a foreign agenda and a means of maximizing profits for western pharmaceutical companies and their collaborators in government, as they wondered why their pressing needs were ignored for some imagined health threat. Further, some participants perceived the new vaccines as a ploy for controlling the population in Muslim nations.

“New vaccines are produced mainly for the profiteering of western pharmaceutical companies and their local collaborators. Let them distribute the money used to buy the vaccines to alleviate poverty and solve our pressing needs. We also sense some foreign agenda to curb the increasing Muslim population” man, 30-year-old.

Participants also referred to conflicting messages on social media regarding the dangers associated with new vaccines, which erode their confidence in these vaccines. Concerns were expressed about the complex technology deployed for vaccine production without ascertaining the long-term effects.

“I am not willing to take the mpox vaccine just yet, as there have been rumors on social media about new vaccines since the COVID-19 outbreak. The conflicting messages make it difficult for us to trust the products. The complicated technology used and uncertainty about their long-term safety is my major worry,” man, 45-year-old.

Some participants opined that since they are not involved with monkeys and other sources of bush meat, mpox is not their problem. They suggested that the mpox vaccine should be administered to those who consume bush meat and deal with monkeys in the southern parts of Nigeria.

“Mpox affects those that eat or sell bush meat. We do not eat bushmeat here nor are we involved in the trade. So, the vaccines should be given to those in other parts of Nigeria that eat monkey meat and deal in bush meat. That´s it,” man, 42-year-old.

## Discussion

We assessed public awareness, perceptions, and predictors of Mpox We assessed public awareness, perceptions, and predictors of mpox vaccine acceptability in Kano metropolis, in northern Nigeria. We found near-universal awareness of mpox, mainly from electronic media (radio, social media, and television) delivering messages on sources, transmission, symptoms, and prevention. However, there were knowledge gaps, misconceptions, and low-risk perceptions. About one-half of respondents were willing to accept the vaccine and this was predicted by sociodemographic (gender, ethnicity, religion), susceptibility disease-related (perceived severity, risk perception), vaccine confidence (vaccine effectiveness), and health-related behavior (adulthood vaccination, and COVID-19 vaccination). Concerns about vaccine safety, rumors, low-risk perception, mistrust in authorities and pharmaceutical companies, and low-felt need fueled vaccine hesitancy.

The high public awareness about mpox mostly from electronic media sources, is similar to reports from south-south Nigeria [26], where over one half (55%) of the respondents mentioned radio/television as sources, followed by HCWs (30.8%) [26]. Similarly, study participants in Nigeria cited government as their source of information [27], while the US public cited online resources and trusted health professionals [28]. Nonetheless, there were misconceptions about the causative agent and transmission, echoing findings from the US [28], Malaysia [29], and China [30]. The routes of transmission of the mpox virus include direct and indirect contact with an infected animal, human secretions or lesions, or contaminated materials, informing the recommendation for self-isolation for 3 weeks [31,32]. The increasing role of social and traditional electronic media and the recent experiences with COVID-19 could explain the heightened public consciousness about diseases with epidemic potential. These channels could be prioritized when addressing knowledge gaps, myths, and misinformation.

The proportion of respondents who considered mpox a serious threat (52.8%) was lower than in Saudi Arabia (63.3%) [33], but higher than in the US (26.4%) [28], and China (62.7%) [32]. However, the proportion of respondents who self-assessed a high risk (3.6%) was much lower than in Saudi Arabia (43.7%) [33], and China (62.7%) [30]. Similarly, the mention of hunters, butchers, and bushmeat dealers as occupational risk groups concurs with reports from parts of Nigeria and Africa [4]. For instance, in the DRC, members of the public identified contact with infected wildlife, peri-domestic animal bites, hunting, and forest visits as mpox risk behaviors. These risk groups should be prioritized for behavioral change communication, including vaccination and the adoption of alternative means of livelihood [4].

Mpox vaccine acceptance in our sample (49.9%) was comparable to reports from Ghana (46.1%) [4], Saudi Arabia (43.7%) [33], and the US (46%) [28], but lower than China (68.8%) [30]. Our figure was also comparable with the global average (43%), and the regional figure for Asia (50%), but lower than that of Europe (70%) [18], the global averages for healthcare workers (63.0%), and the LGBTI community (84.0%) [18]. Apart from variations in study timing, population, and methods, differences in disease burden, knowledge, risk perception, trust in authorities, and perceived benefits of mpox vaccination could explain the disparity in vaccine acceptance. These modulators could inform strategies for enhancing vaccine uptake through appropriate risk communication and public engagement.

The reasons for mpox vaccine hesitancy fit into four of the categories of the 5C model, including vaccine confidence (doubts about vaccine effectiveness, safety, and policymakers´ motivation), complacency (perceived risk and threat level), the risk-benefit calculation (information on benefits and effects of anti-vaccination campaigns, rumors) and vaccination being a collective community responsibility (willingness to protect others by getting vaccinated). The last category is illustrated by participants´ suggestions that vaccination be limited to more endemic settings and high-risk occupations [34]. Constraints to vaccination which constitute the last ‘C’ could surface after the rollout of the mpox vaccination program. Our findings are similar to reports from other parts of Nigeria [26,35], Asia [30]. Europe and the United States [18,28]. Apart from study methods, motivations for vaccination could be influenced by philosophical stance, and community adhesion. In addition to the lingering effects of the COVID-19 anti-vaccination messages on public psyche, vaccination fatigue, low felt need, and perceived neglect of public priorities could underlie some of the resistance towards vaccination. Evidence-based steps are essential to counter misinformation, rebuild public trust, and vaccine confidence.

The predictive role of gender was also reported in Ghana [36], and could operate through rumors linking vaccines to infertility, and doubts about maternal and fetal safety during pregnancy and breastfeeding [37]. The influence of ethnicity could be related to the long-held belief that vaccination is a ploy for targeted population control and mistrust about the motives of authorities and pharmaceutical companies. Most religions encourage vaccination for protection against diseases. However, some religious leaders constitute obstacles, as was the case with polio vaccination campaigns in northern Nigeria [38]. Similarly, perceived disease severity and risk perception have been reported to influence health behavior including vaccination decisions [28,30]. Vaccination during adulthood, including COVID-19 vaccination [36], are indicators of a positive disposition towards vaccination [28,30].

Customized behavioral change communication interventions are necessary to address misconceptions and the low-risk perception. Before considering for the rollout of the mpox vaccination in Nigeria, appropriate risk communication and strategies to address the root causes of vaccine hesitancy should be implemented. This should include rumor surveillance, and counter-messaging to build public confidence. Appropriate messages should be conveyed through traditional and social media [27,28], with consideration for gender and socio-cultural peculiarities.

This mixed methods study is among the first to assess the acceptability of the mpox vaccine in an endemic setting in northern Nigeria. The face-to-face survey ensured the inclusion of less tech-savvy individuals, while the in-depth interviews provided detailed descriptions of motives for vaccine acceptance and reasons for hesitancy in participants´ voices. Nonetheless, there is a need for caution when generalizing the findings given the cultural variations that exist across Nigeria. Future studies should assess the situation in other locations, including rural and semi-urban settings.

## Conclusion

We found near-universal awareness of the mpox vaccine coupled with misconceptions and low-risk perception of mpox acquisition among respondents. Vaccine acceptance was sub-optimal and influenced by sociodemographic, vulnerability, and health behavior-related factors. Risk communication, strategic community engagement, and socio-behavioral interventions will help to address misconceptions, safety concerns, and vaccine hesitancy, and thereby increase vaccine uptake in similar settings.

### What is known about this topic

- Outbreaks of Mpox have been recently reported in areas outside of the endemic regions of the world;
- Little has been documented about public perceptions and acceptance of the Mpox vaccine in endemic countries.

### What this study adds

- We found near-universal awareness and suboptimal acceptance of the Mpox vaccine among adult men and women resident in a large Northern Nigerian city;
- Acceptance of the Mpox vaccine was influenced by sociodemographic, vulnerability, and health behavior-related factors.
